# Weather Changes to the Old

**Published:** 1874-01

**Authors:** 


### WEATHER CHANGES TO THE OLD.

UP to thirty years of age the system bears changes of temperature better than later in life. All know the injurious nature of sudden changes from a colder to a warmer air. Observation shows that while there is one death from such sudden change, among persons about thirty years of age, there will be two deaths among an equal number at thirty-nine, four deaths among an equal number nine years later—that is, at forty-eight; eight deaths at fifty-seven, sixteen at sixty-six, thirty-two at seventy-five, sixty-four at eighty-four; hence there is a rapidly increasing necessity, after "three score" of guarding against exposure to sudden changes of weather; while one dies at thirty, thirty-two die at seventy-five. It is said of the Duke of Wellington that at four score it required him to keep his room so warm, in order to render him comfortable, that few persons could remain in it with any degree of satisfaction longer than a very few moments at a time, and that he always put his head out of the window on rising in the morning, to determine by his feelings the temperature of the air, and then would order a coat to be brought to him adapted to the temperature. It was by such carefulness that he was able to reach a good old age.

No one, after "three score," can afford to neglect these little precautions. It cannot be done with impunity. It is for the want of it that so many persons, after that age, in apparent health, are hurried to the grave in a few days from pneumonia, known commonly as inflammation of the lungs.

It would answer a valuable purpose if all old and frail persons would have a permanent thermometer outside the chamber window, and one inside, so as to determine every morning the difference between the outer and inner temperature. A difference of twenty degrees or more, especially if there is much wind, imperatively demands a warmer dress for the outside, and not to be changed to a thinner material until next morning.

